# Supplementary material for: Integrase Strand Transfer Inhibitors Are Effective Anti-HIV Drugs
Source: Viruses. 2021 Jan 29;13(2):205. doi: 10.3390/v13020205 (PMC7912079; doi:10.3390/v13020205)
Supplement: Supplementary file 1 [file viruses-13-00205-s001.pdf]

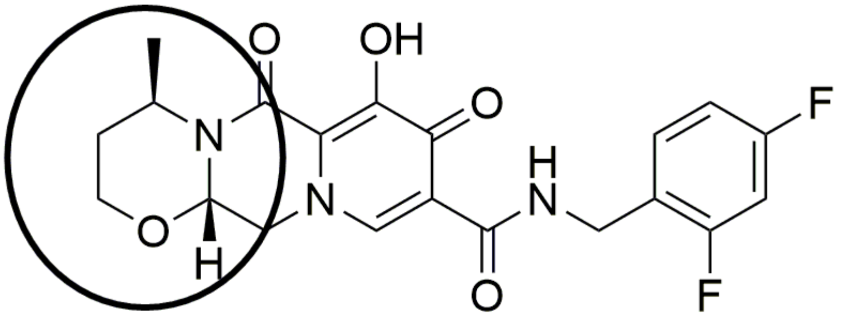

Dolutegravir (DTG)

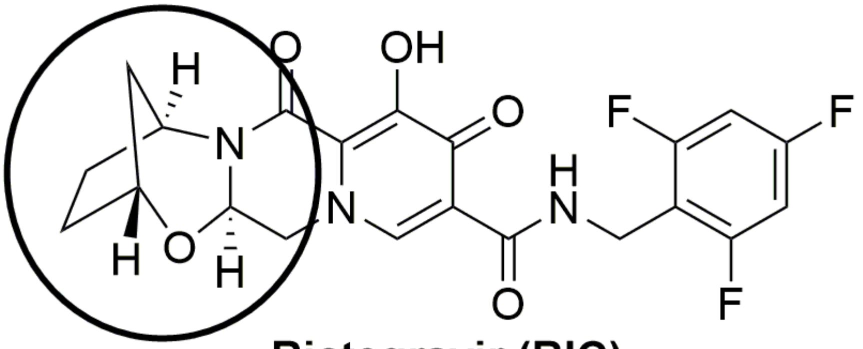

Bictegravir (BIC)

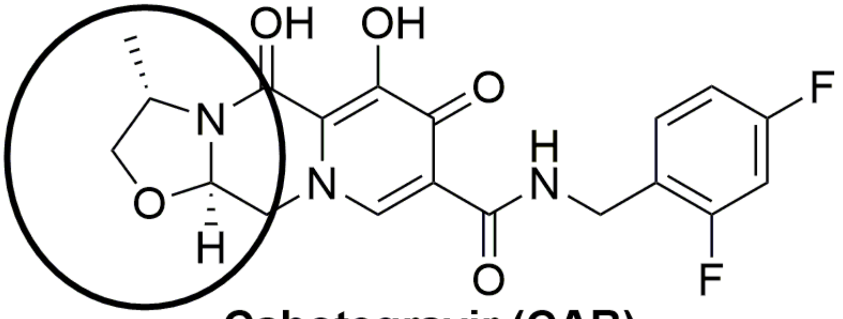

Cabotegravir (CAB)

Supplementary Figure 1. Chemical structure of the “A” ring of the second generation INSTIs. Chemical structures of the second generation INSTIs are shown with their respective “A” rings surrounded with a black circle.

Table S1. Current recommendations for HIV-1 treatments with INSTIs. The recommended INSTI treatment strategies for most ARV naïve or ARV-experienced, INSTI native HIV-1 patients are shown at the top of the table. The recommended treatment strategies for most ARV naïve or ARV-experienced, INSTI-naïve in certain clinical settings are given in the middle panel. The treatment strategies for most suppressed HIV-1 patients undergoing maintenance therapy are shown in the bottom panel.

INSTI Treatment Strategies for Most ARV Naïve or ARV-Experienced, INSTI Naïve, HIV-1 Patients			
Name	ARV Drugs Formulated into Fixed Dose Combinations	Dosing Recommendation	Adverse Events
Biktarvy	Bictegravir (50 mg)/tenofovir alafenamide (25 mg)/emtricitabine (200 mg)	One tablet once daily	Diarrhea, nausea, headache, weight gain
Triumeq	Dolutegravir (50 mg)/abacavir (600 mg)/lamivudine (300 mg)	One tablet once daily	Insomnia, headache, weight gain, hepatotoxicity, depression, slightly increased risk of neural tube defects in infants
Dovato*	Dolutegravir (50 mg)/lamivudine (300 mg)	One tablet once daily	Insomnia, headache, weight gain, hepatotoxicity, depression, slightly increased risk of neural tube defects in infants
Isentress plus Truvada	Raltegravir (400mg)/tenofovir disoproxil fumarate (300 mg)/emtricitabine (200 mg)	Two tablets daily	Nausea, weight gain, insomnia, depression, rash
Isentress HD plus Truvada	Raltegravir (600mg)/tenofovir disoproxil fumarate (300 mg)/emtricitabine (200 mg)	Two tablets once daily	Nausea, weight gain, insomnia, depression, rash
*only for individuals with HIV RNA ≤500,000 copies/mL			

INSTI Treatment Strategies for Most ARV Naïve or ARV-Experienced, INSTI-Naïve HIV-1 Patients in Certain Clinical Settings			
Name	ARV Drugs Formulated into Fixed Dose Combinations	Dosing Recommendation	Adverse Events
Genvoya	Elvitegravir (150 mg)/cobicistat (150 mg)/tenofoviar alafenamide (10 mg)/emtricitabine (200 mg)	One tablet once daily with food	Nausea, diarrhea, depression
Stribild	Elvitegravir (150 mg)/cobicistat (150 mg)/tenofovir disoproxil fumarate (300 mg)/emtricitabine (200 mg)	One tablet once daily with food	Nausea, diarrhea, depression

INSTI Treatment Strategies for Most Suppressed HIV-1 Patients Undergoing Maintenance Therapy			
Name	ARV Drugs Formulated into Fixed Dose Combinations	Dosing Recommendation	Adverse Events
Dovato*	Dolutegravir (50 mg)/lamivudine (300 mg)	One tablet once daily	Insomnia, headache, weight gain, hepatotoxicity, depression, slightly increased risk of neural tube defects in infants
Juluca	Dolutegravir (50 mg)/rilpivirine (25 mg)	One tablet once daily with a meal	Insomnia, headache, weight gain, hepatotoxicity, depression, slightly increased risk of neural tube defects in infants
Isentress HD plus Truvada	Raltegravir (600mg)/tenofovir disoproxil fumarate (300 mg)/emtricitabine (200 mg)	Two tablets once daily	Nausea, weight gain, insomnia, depression, rash
Triumeq	Dolutegravir (50 mg)/abacavir (600 mg)/lamivudine (300 mg)	One tablet twice daily	Insomnia, headache, weight gain, hepatotoxicity, depression, slightly increased risk of neural tube defects in infants
Biktarvy	Bictegravir (50 mg)/tenofovir alafenamide (25 mg)/emtricitabine (200 mg)	One tablet once daily	Diarrhea, nausea, headache, weight gain
